# Partial and complete trisomy 14 mosaicism: clinical follow-up, cytogenetic and molecular analysis

**DOI:** 10.1186/s13039-014-0065-8

**Published:** 2014-09-25

**Authors:** Consuelo Salas-Labadía, Esther Lieberman, Roberto Cruz-Alcívar, Pilar Navarrete-Meneses, Samuel Gómez, Consuelo Cantú-Reyna, Karin Buiting, Carola Durán-McKinster, Patricia Pérez-Vera

**Affiliations:** Departamento de Genética Humana, Laboratorio de Cultivo de Tejidos, Instituto Nacional de Pediatría, Insurgentes Sur 3700-C, México, DF C.P. 04530 Mexico; Departamento de Genética Humana, Instituto Nacional de Pediatría, México, DF Mexico; GENOMI-k, Monterrey, NL Mexico; Institut für Humangenetik Universitätsklinikum, Essen, Germany; Servicio de Dermatología, Instituto Nacional de Pediatría, México, DF Mexico

**Keywords:** Marker chromosome 14, Trisomy 14 mosaicism, Deletion 14q11.2, Microarray analysis

## Abstract

**Background:**

Trisomy 14 mosaicism is a rare chromosomal abnormality. It is associated with multiple congenital anomalies. We report a 15 year-old female with an unusual karyotype with three cell lines: 47,XX,+mar/47,XX,+14/46,XX. At six months old she had short stature, cleft palate, hyperpigmented linear spots in arms and legs and developmental delay. At present, she has mild facial dysmorphism and moderate mental retardation.

**Methods:**

Cytogenetic analysis was performed in peripheral blood lymphocytes and in the light and dark skin following standard methods. DNAarray – Oligo 180 k was carried out using Agilent Technologies and FISH analysis was accomplished using DNA BACs probes to confirm the result obtained by DNAarray. Methylation-Specific PCR (MS-PCR) of the *MEG3* promoter and microsatellite analysis were performed.

**Results:**

Microarray analysis confirmed partial trisomy 14 mosaicism; the marker chromosome was found to be from chromosome 14, the result was confirmed with FISH. Methylation (14q32.3) and microsatellite (14q11-14q32.33) analysis were carried out and UPD was discarded. The global result was: mos 47,XX,+del(14)(q11.2)[45]/47,XX,+14[10]/46,XX[45].

**Conclusions:**

This is a unique case because of the coexistence of two abnormal cell lines, including one with +14 and another with +del(14)(q11.2). To our knowledge, only three patients have been reported with trisomy 14 and another abnormal cell line. The array analysis identified the marker chromosome and characterized the breakpoint. The del(14)(q11.2) does not seem to be related to any particular phenotypic characteristic of the patient; the clinical features of our patient observed until now, can be attributed to trisomy 14 mosaicism. Nevertheless, we cannot discard the manifestation of new symptoms related to her karyotype in the future.

**Electronic supplementary material:**

The online version of this article (doi:10.1186/s13039-014-0065-8) contains supplementary material, which is available to authorized users.

## Background

Trisomy 14 mosaicism is a rare chromosomal abnormality with an incidence of 3:1 females compared to males and is associated with multiple congenital anomalies [1-3]. The most common clinical characteristics (Table 1) are growth and psychomotor retardation, dysmorphic craniofacial features such as broad nose, abnormal or low-set ears, micrognathia, cleft or highly arched palate, short neck, congenital heart and genitourinary abnormalities [3,4]. Other features reported are hypertelorism, body asymmetry and abnormal skin pigmentation [1,2].

**Table 1:** **Clinical features of patients with trisomy 14**, **mosaicism and sSMC 14**

**Clinical features**	**% of cases with mos trisomy 14** ^*^	**% of cases with sSMC 14** ^**^	**Our patient**
Growth retardation	70^1^	45	+
Developmental/Mental delay	70^2^	27	+
Hypotonia	24^3^	18	-
Microcephaly	25^4^	9	+
Hearing problems	20^5^	9	+
Eye abnormalities	75^6^	9	-
Mouth abnormalities	80^7^	-	
Heart defect	60^8^	9	+
Hip problems	7.5^9^	9	
Extremities anomalies	58^10^	27	+
Genitourinary abnormalities	38^11^	9	-
Pigmentary skin lesions	38^12^	-	+

mos = mosaic, sSMC = small supernumerary marker chromosome.

^*^Supplementary online reference list.

**References 1)** 1-4,6,8-10,12,16,17a,17b,20,22,24,26,30-33a-e; **2)** 2,3,5,8-12,15,17a-b,18,20,24,27,31-34; **3)** 2,8,13,30,33a,33b,33d,33e; **4)** 1,3,5-8,10,15,17a,31; **5)** 5,10,17b,24,33b-e; **6)** 1-13,16,17a-b,20,22-25,27,31,32,33a-e,35; **7)** 1-4,6-11,13,15-19,21,22,24-33a-e; **8)** 1,3,4,6,9-13,16-17a-b,20,21,23-26,29,32,33b,33d,33e,35; **9)** 13,15,33e; **10)** 1,2,4-7,9,11,13,16,17b,21,26,27,30,31,33a-e,35; **11)** 4,6,7,11,13,16,17a,20,25,30,33a-b,33d-e,34; **12)** 2,3,6,8,10,15-17a,20,22-24,31,32,34; from online supplementary list (Additional file 1)**; a,b,c,d,e**: Patient 1,2,3,4,5.

^**^From http://ssmc-tl.com/sSMC.html [accessed 05/09/2014].

To our knowledge, 40 liveborn with trisomy 14 mosaicism have been reported (Additional file 1). The most frequent cytogenetic finding is the presence of an extra chromosome 14 in mosaic (24 cases, 60%), followed by occurrence of an isochromosome 14 (9 cases, 22.5%), or a robertsonian or non-homologous reciprocal translocation involving chromosome 14 (5 cases, 12.5%), or a ring 14 (2 cases, 5%), which originates isodicentric clones involving chromosome 14 (Additional file 1).

Complete trisomy 14 is highly lethal for the early embryo; liveborns have been reported only in mosaicism [3]. The mechanisms for acquiring the extra chromosome 14 are: a) maternal non-disjunction (72%: 36% meiosis I (MI) and 36% meiosis II (MII); b) paternal non-disjunction (20%: 100% MII); and c) post-zygotic errors (8%) [5].

Chromosome 14 mosaicism cannot only arise from gaining the complete chromosome, but also from small supernumerary marker chromosomes (sSMCs) producing partial trisomy [6]. sSMCs constitute a morphologically heterogeneous group of structurally abnormal chromosomes that cannot be characterized by conventional banding cytogenetics [7]. In general, they are equal in size or smaller than chromosome 20 of the same metaphase spread [6,7]. sSMCs can be recognized as inverted duplicated chromosomes, minute chromosomes, ring or deleted chromosomes, being the mechanism of formation different in each case. sSMCs cell lines have been reported in mosaic with 46 normal chromosomes and numerically abnormal or structurally abnormal balanced karyotypes [6,8]. Phenotypes associated with sSMCs are highly variable (Table 1). Besides the chromosomal imbalance there are other factors that influence phenotype, such as the level of mosaicism [6]. Mosaicism of sSMC 14 has been reported in about 32 patients; in some cases presenting with clinical features that can include: short stature, mental retardation, microcephaly, hypoplastic alae nasi, midface hypoplasia, exophthalmos, bilateral myopia, retinal microangiopathy, cleft lip and palate, congenital malformation of pancreas, secondary cardiomyopathy, hypogonadism and club feet (Table 1) [8,9]. Furthermore, trisomy 14 in mosaic can also be influenced by uniparental disomy (UPD). Reports of maternal UPD14 phenotype include intrauterine growth retardation, precocious puberty, hypotonia at birth, feeding difficulties in early infancy, short stature, small hands and feet, scoliosis, mild developmental delay and minor facial dysmorphism [10,11]. Paternal UPD14 is less frequent and more severe; it causes bell-shaped thoracic deformity secondary to the presence of “coat-hanger” ribs, polyhydramnios, abdominal wall defects and dysmorphic features [11,12].

We report the case of a female with trisomy 14 in mosaic with two abnormal cell lines, one with +14 chromosome, and another with sSMC. She has been analyzed by DNA oligoarray and FISH to establish the sSMC origin and to characterize the abnormality and exact breakpoint. A clinical correlation with these abnormalities and a follow-up during 15 years are also included.

## Case presentation

**Figure 1:**
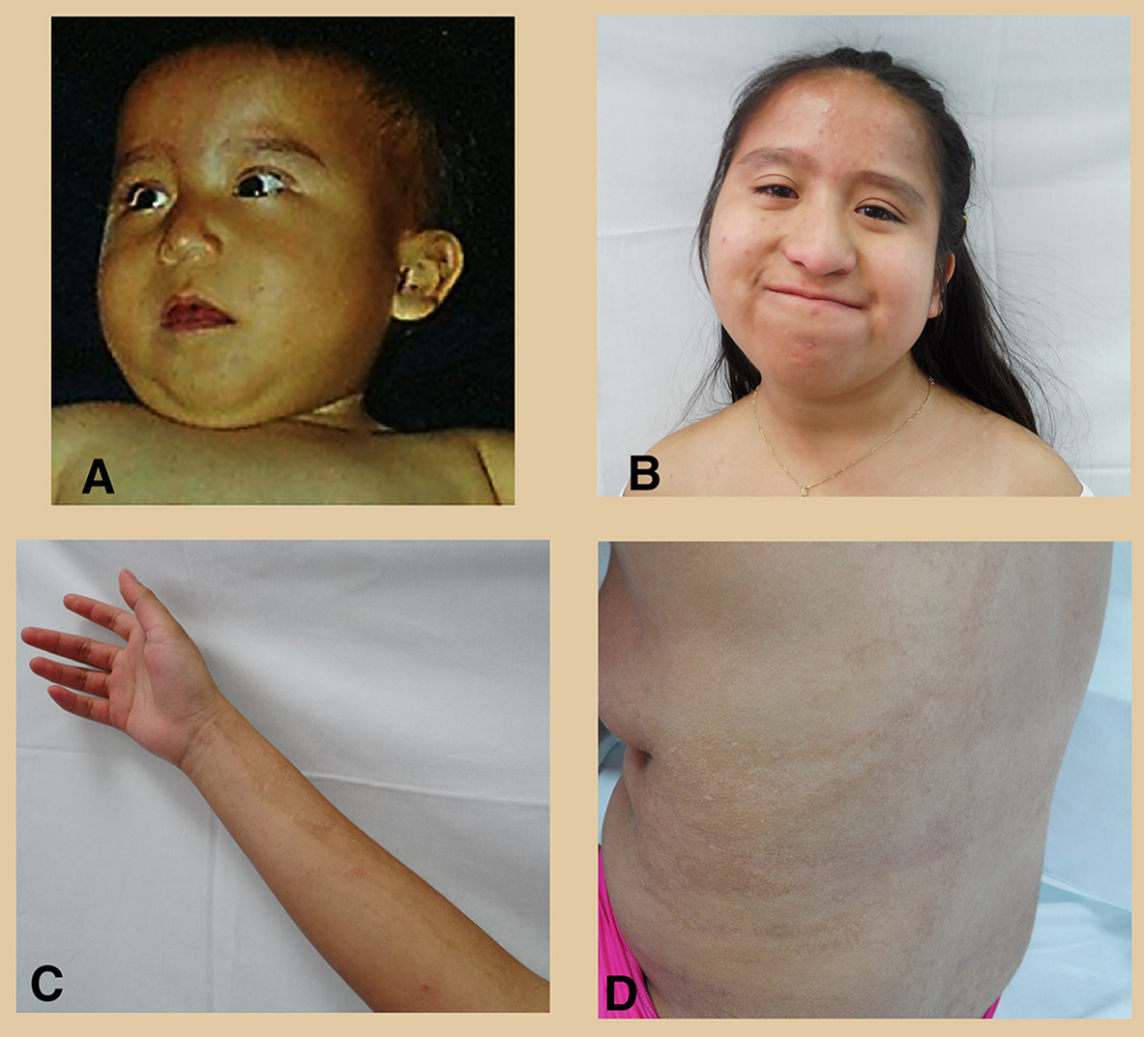
**Photographs of the patient at different ages showing face asymmetry and hyperpigmented lines A) Patient at 6 months; B) At 15 years old.** Note the pigmentary changes including a well-delimited hyperpigmented pattern in: **C)** Arm and **D)** Dorsal region.

The patient is a 15 year-old female, second child of healthy non-consanguineous parents who had one previous miscarriage. At six months of age (Figure 1A), she had short stature, cleft palate, face asymmetry, hyperpigmented linear macules in anterior and posterior dorsum, arms and legs following the lines of Blaschko, developmental delay, esophageal reflux, lumbar scoliosis and hip dysplasia. At present (Figure 1B), she has mild facial dysmorphism with face asymmetry, moderate mental retardation, bilateral conductive deafness, nocturnal enuresis and maintains hyperpigmented linear macules in dorsum and limbs (Figures 1C-D). She has undergone palate closure, Nissen funduplication and hip myotomy. MRI shows arachnoid cysts.

### Cytogenetic and molecular analysis

#### Lymphocyte and fibroblast cultures

Cytogenetic analysis was performed in peripheral blood lymphocytes following standard methods [13]. G-banded metaphases were analyzed and interpreted according to the International System for Human Cytogenetic Nomenclature 2013 [7]. Fresh biopsies were obtained from dark and light skin. Fibroblasts were cultured with complete-Amniomax medium (Gibco, USA) for 10-15 days. Re-seeded cells on glass coverslips were incubated with colcemid (10 mg/ml; Gibco, USA) for 20 min and harvested to obtain metaphases. G-banded metaphases were obtained and analyzed following the same criteria as for lymphocytes.

### DNA extraction and DNAarray – Oligo 180 k

DNA for array was isolated from peripheral blood, using the Qiagen Kit according to the manufacturer’s instructions. DNA sample with absorbance ratios between 1.8 and 2.0 was used. DNAarray – Oligo 180 k was carried out using Agilent Technologies (Santa Clara, CA), which contains 170,000 probes, distributed between coding and non-coding human genome sequences (hg18, UCSC) with a ~16 kb resolution. The sample was evaluated and specific assessment was made in relation to the change in the number of copies that involves >6 probes in all regions of the genome including pericentromeric and subtelomeric regions, and in areas with known diagnostic relevance of microduplication/microdeletion syndromes.

### FISH

FISH analysis was accomplished in lymphocytes using DNA BACs probes for 14q11.2 (RP11-232H9, spectrum green), and 14q32.33 (CTD-2313E3, spectrum orange). Hybridization was performed according to reported methods [14]. Analysis was performed using a microscope AXIO ImagerMI (Zeiss; Jena, Germany) and the images were captured and analyzed with the ISIS software (Meta Systems, Altlussheim, Germany). A total of 52 metaphases were reviewed.

### Parental origin analysis

#### Methylation-Specific PCR (MS-PCR) of the *MEG3* Promoter and microsatellite analysis

Considering the patient’s karyotype and phenotypic features, the bisulfite modification of genomic DNA was performed for UPD14 analysis. DNA samples from the patient and both parents were analyzed. Methylation specific PCR reactions with maternal/paternal oligonucleotide primers for the differentially methylated region of the *MEG3* promoter on chromosome 14q32 were performed as previously described [15]. Normal samples were included as controls. Microsatellite analysis was performed using markers localized from 14q11 through 14q32.33 (*D14S597, D14S290, D14S81, D14S267, D14S250, D14S78, D14S1006, D14S1010*) [10]. Fragment length analysis was performed on a 3100 genetic analyser (Applied Biosystems) and interpreted using the GeneMarker software (Softgenetics).

### Results

**Figure 2:**
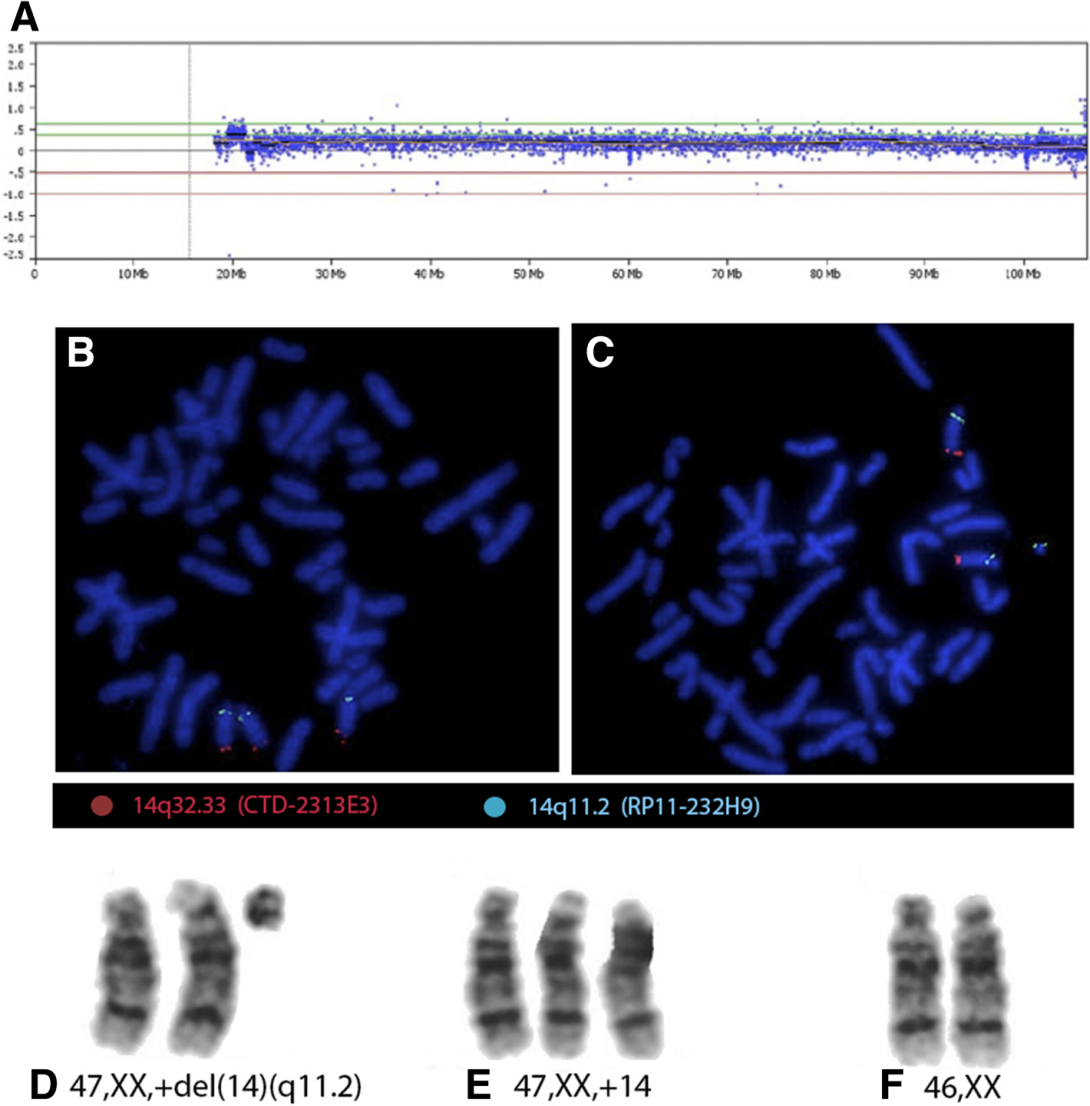
**Cytogenomic analysis A) Microarray analysis shows a partial trisomy 14 of the proximal region between 19.5 Mb and 21.3 Mb (arr 14q11.1q11.2(18,127,052-19,927,052)** **x** **2~3).** FISH with DNA BACs probes for 14q11.2 spectrum green and 14q32.33 spectrum orange shows: **B)** Metaphase with whole trisomy 14, each chromosome 14 with one green and one orange signals; **C)** Metaphase with del(14)(q11.2) lacking 14q32.33 orange signal. GTG-banded partial karyotype showing: **D)** +del(14)(q11.2) **E)** trisomy 14 and **F)** normal diploid chromosomes 14.

Cytogenetic analysis in lymphocytes revealed three cell lines: mos 47,XX,+mar[45]/47,XX,+14[10]/46,XX[45]. In fibroblasts from both types of skin, two cell lines were detected: Light skin: mos 47,XX,+mar[7]/46,XX[8]; dark skin: mos 47,XX,+mar[12]/46,XX[14]. The microarray revealed a partial trisomy 14 mosaicism (arr 14q11.1q11.2(18,127,052-19,927,052)x2~3), identifying the marker chromosome as chromosome 14 (1.8 MB), resulting in partial trisomy of the proximal region between 19.5 Mb and 21.3 Mb (Figure 2A). FISH revealed the presence of three cell lines: 55.8% normal, 9.6% trisomic (+14) and 34.6% with a deleted chromosome 14 (sSMC14) (Figures 2 B-C). The final result was: mos 47,XX,+del(14)(q11.2)[45]/47,XX,+14[10]/46,XX[45] (Figures 2D-F). Methylation and microsatellite analysis did not reveal evidence of UPD 14. Parental karyotypes were normal.

## Conclusions

This is a unique case because of the presence of an abnormal cell line +del(14)(q11.2) additional to the trisomy 14. To our knowledge, there are no other similar cases reported (Additional file 1). *Becerra-Solano*, *et al.* reported a patient with a 45,X cell line besides the trisomy 14, however these are two non-cytogenetically related clones [16]. There are two cases reporting more than one abnormal cell line involving chromosome 14, nevertheless they lack the supernumerary chromosome 14 [17,18].

For the present case, the mechanism of origin proposed regarding a cell line with supernumerary marker coexisting with trisomic and disomic cell lines provide evidence for trisomy rescue. The zygote may be originated as a trisomy by a meiosis I error, a disomic gamete forms a trisomic zygote and trisomic rescue take place generating the 3 cell lines observed: 47,XX,+del(14)(q11.2)/47,XX,+14/46,XX [19-21].

The DNA array analysis identified the marker chromosome and characterized the breakpoint. Although patients with del(14)(q11.2) present non-specific clinical features and 60% of them lack clinical findings; a search of genes comprised in this region was performed using the OMIM database for establishing a phenotype-genotype correlation. We found genes located on 14q11.2 involved in cardiomyopathy (Myosin heavy polypeptide 6 cardiac muscle alpha, *MYH6*; Myosin heavy polypeptide 7 cardiac muscle beta *MYH7*), neural deafness (Deafness autosomal dominant 53, *DFNA53*; Deafness autosomal recessive 5, *DFNB5*) and retinal degeneration (Retinal degeneration autosomal recessive clumped pigment type, *NRL*). These features were not detected in the patient, although they are reported in other cases with trisomy 14 or sSMC14 (Table 1). In this case, sSMC14 does not seem to be related to any particular feature of the patient. Methylation and microsatellite analysis did not reveal UPD.

Considering the results obtained from the gene search and maternal UPD analysis, the clinical features of our patient observed until now, could be attributed to trisomy 14 in mosaic. Nevertheless, we cannot discard the manifestation of new symptoms related to her karyotype in the future. A follow-up of this patient must be performed in order to evaluate this possibility.

### Consent

Written informed consent was obtained from the patient’s parents for publication and accompanying images of this case report. A copy of the written consent is available for review by the Editor-in-Chief of this journal.

## Additional file

Additional file 1
**Supplementary online references list.**
