# Supplementary material for: Partial and complete trisomy 14 mosaicism: clinical follow-up, cytogenetic and molecular analysis
Source: Mol Cytogenet. 2014 Sep 25;7:65. doi: 10.1186/s13039-014-0065-8 (PMC4180134; doi:10.1186/s13039-014-0065-8)
Supplement: Additional file 1 — Supplementary online references list. [file 13039_2014_65_MOESM1_ESM.docx]

1. Rethoré MO, Couturier J, Carpentier S, Ferrand J, Lejeune J. [Mosaic 14 trisomy in a female child with multiple abnormalities]. Ann Génétique 1975,18:71–4.

2. Martin AO, Ford MM, Khalil NT, Turk KB, Macintyre MN. 46,XX/47XX, + 14 mosaicism in a liveborn infant. J Med Genet 1977,14:214–8.

3. Johnson VP, Aceto T Jr, Likness C. Trisomy 14 mosaicism: case report and review. Am J Med Genet 1979,3:331–9.

4. Turleau C, de Grouchy J, Cornu A, Turquet M, Millet G. [Mosaic trisomy 14 due to an iso dicentric chromosome (author’s transl)]. Ann Génétique 1980,23:238–40.

5. Jenkins MB, Kriel R, Boyd L. Trisomy 14 mosaicism in a translocation 14q15q carrier: probable dissociation and isochromosome formation. J Med Genet 1981,18:68–71.

6. Dallapiccola B, Ferranti G, Giannotti A, Novelli G, Pasquini L, Porfirio B. A live infant with trisomy 14 mosaicism and nuclear abnormalities of the neutrophils. J Med Genet 1984,21:467–70.

7. Ozawa N, Xu ZD, Soh K, Takabayashi T, Sato S, Yajima A, et al. A case of mosaic trisomy 14 due to an isochromosome, i(14q). Jinrui Idengaku Zasshi Jpn J Hum Genet 1984,29:69–76.

8. Pangalos C, Velissariou V, Ghica M, Liacacos D. Ring-14 and trisomy 14q in the same child. Ann Génétique 1984,27:38–40.

9. Petersen MB, Vejerslev LO, Beck B. Trisomy 14 mosaicism in a 2 year old girl. J Med Genet 1986,23:86–8.

10. Fujimoto A, Lin MS, Korula SR, Wilson MG. Trisomy 14 mosaicism with t(14;15)(q11;p11) in offspring of a balanced translocation carrier mother. Am J Med Genet 1985,22:333–42.

11. Kaplan LC, Wayne A, Crowell S, Latt SA. Trisomy 14 mosaicism in a liveborn male: clinical report and review of the literature. Am J Med Genet 1986,23:925–30.

12. Lipson MH. Trisomy 14 mosaicism syndrome. Am J Med Genet 1987,26:541–4.

13. Cheung SW, Kolacki PL, Watson MS, Crane JP. Prenatal diagnosis, fetal pathology, and cytogenetic analysis of mosaic trisomy 14. Prenat Diagn 1988,8:677–82.

14. Wegner RD, Hohle R, Karkut G, Sperling K. Trisomy 14 mosaicism leading to cytogenetic discrepancies in chorionic villi sampled at different times. Prenat Diagn 1988,8:239–43.

15. Cantú ES, Thomas IT, Frias JL. Unusual cytogenetic mosaicism involving chromosome 14 abnormalities in a child with an MR/MCA syndrome and abnormal pigmentation. Clin Genet 1989,36:189–95.

16. Vachvanichsanong P, Jinorose U, Sangnuachua P. Trisomy 14 mosaicism in a 5-year-old boy. Am J Med Genet 1991,40:80–3.

17. Fujimoto A, Allanson J, Crowe CA, Lipson MH, Johnson VP. Natural history of mosaic trisomy 14 syndrome. Am J Med Genet 1992,44:189–96.

18. Antonarakis SE, Blouin JL, Maher J, Avramopoulos D, Thomas G, Talbot CC Jr. Maternal uniparental disomy for human chromosome 14, due to loss of a chromosome 14 from somatic cells with t(13;14) trisomy 14. Am J Hum Genet 1993,52:45–52.

19. Lambert I, Kemp J, Jackson J, Joyce H, Mann S, Kan A, et al. Prenatal diagnosis and post-mortem study of a fetus with mosaic trisomy 14 due to a dic(14)(p11). Prenat Diagn 1994,14:507–10.

20. Iglesias A, McCurdy LD, Glass IA, Cotter PD, Illueca M, Perenyi A, et al. Mosaic trisomy 14 with hepatic involvement. Ann Génétique 1997,40:104–8.

21. Sepulveda W, Monckeberg MJ, Be C. Twin pregnancy discordant for trisomy 14 mosaicism: prenatal sonographic findings. Prenat Diagn 1998,18:481–4.

22. Tunca Y, Wilroy RS, Kadandale JS, Martens PR, Gunther WM, Tharapel AT. Hypomelanosis of ito and a “mirror image” whole chromosome duplication resulting in trisomy 14 mosaicism. Ann Génétique 2000,43:39–43.

23. Kuwahara RT, Henson T, Tunca Y, Wilroy SW. Hyperpigmentation along the lines of Blaschko with associated chromosome 14 mosaicism. Pediatr Dermatol 2001,18:360–1.

24. Lynch MF, Fernandes CJ, Shaffer LG, Potocki L. Trisomy 14 mosaicism: a case report and review of the literature. J Perinatol 2004,24:121–3.

25. Witters I, Moerman P, Fryns J-P. First-trimester scan in trisomy 14 mosaicism. Prenat Diagn 2004,24:573–4.

26. Kunst G, Gillbe C. General anesthesia for cardiac catheterization in a child with trisomy 14 mosaicism. Anesth Analg 2005,100:1860.

27. Tzoufi M, Kanioglou C, Dasoula A, Asproudis I, Tsatsoulis A, Sismani C, et al. Mosaic trisomy r(14) associated with epilepsy and mental retardation. J Child Neurol 2007,22:869–73.

28. Gerard-Blanluet M, Pipiras E, Levaillant JM, Joye N, Koubi V, Kanafani S, et al. Prenatal detection of Pierre Robin sequence with deletion Xp and additional trisomy 14q by telomere screening. Prenat Diagn 2007,27:1062–3.

29. Wang J-C, Li CF, Shaw DRS. Prenatally diagnosed mosaic trisomy 14q with omphalocele. Prenat Diagn 2007,27:1260–1.

30. Merritt TA, Natarajan G. Trisomy 14 Mosaicism: a case without evidence of neurodevelopmental delay and a review of the literature. Am J Perinatol 2007,24:563–6.

31. Becerra-Solano LE, Arnaud-Lopez L, Diaz-Rodriguez M, Mantilla-Capacho JM, Nastasi-Catanese JA, Ortiz-Aranda M, et al. First case reported of Turner syndrome and trisomy 14 chromosomal mosaicism in a patient. Clin Dysmorphol 2008,17:27–30.

32. Von Sneidern E, Lacassie Y. Is trisomy 14 mosaic a clinically recognizable syndrome?--case report and review. Am J Med Genet A 2008,146A:1609–13.

33. Shinawi M, Shao L, Jeng LJB, Shaw CA, Patel A, Bacino C, et al. Low-level mosaicism of trisomy 14: phenotypic and molecular characterization. Am J Med Genet A 2008,146A:1395–405.

34. McGaughran J, Stevens R, Blond A, Perry C. Nasal encephalocele in a child with mosaic trisomy 14. Clin Dysmorphol 2009,18:164–5.

35. Choi JH, Choi YJ, Kim SY. Congenital ocular anomaly in an infant with trisomy 14 mosaicism. Korean J Ophthalmol KJO 2012,26:316–8.
